# Hyperparathyroidism, Serum Phosphorus and Dietary Intake in Hemodialysis Patients: Is There a Novel Relationship?

**DOI:** 10.3390/ijms25042006

**Published:** 2024-02-07

**Authors:** Cristina Garagarza, Ana Valente, Cátia Queirós, Inês Pastor Neto, Joana Sebastião, Melanie Gomes, Aníbal Ferreira

**Affiliations:** 1 Nutrition Department, Nephrocare, 1750 Lisboa, Portugal; ana.valente@freseniusmedicalcare.com (A.V.); catia_silvaqueiros@outlook.com (C.Q.); ines.neto@freseniusmedicalcare.com (I.P.N.); joana.sebastiao@freseniusmedicalcare.com (J.S.); melanie.gomes@freseniusmedicalcare.com (M.G.); 2 Laboratório de Nutrição, Faculdade de Medicina, Universidade de Lisboa, 1649 Lisboa, Portugal; 3 Serviço de Nefrologia, Hospital de Curry Cabral—Centro Hospitalar Universitário de Lisboa Central, 1069 Lisboa, Portugal; 4 NOVA Medical School, Faculdade de Ciências Médicas, Universidade Nova de Lisboa Centro Clínico Académico de Lisboa, 1169 Lisboa, Portugal

**Keywords:** parathyroid hormone, food intake, nutritional status, dialysis

## Abstract

The management of hyperparathyroidism (intact parathyroid hormone (iPTH) serum levels > 585 pg/mL), frequently focuses on the appropriate control of mineral and bone markers, with the decrease in serum and dietary phosphorus as two of the targets. We aimed to investigate the association between iPTH, serum phosphorus levels and dietary intake. This was a cross-sectional, multicenter, observational study with 561 patients on hemodialysis treatment. Clinical parameters, body composition and dietary intake were assessed. For the analysis, patients were divided into three groups: (a) iPTH < 130, (b) iPTH between 130 and 585 and (c) iPTH > 585 pg/mL. The association between PTH, serum phosphorus and dietary intake was analyzed using linear regression models. In the whole sample, 23.2% of patients presented an iPTH > 585 pg/mL. Patients with higher iPTH levels were those with longer HD vintage and lower ages, higher serum phosphorus, serum calcium, Ca/P product, albumin and caffeine intake, and a lower dietary intake of phosphorus, fiber, riboflavin and folate. Higher serum phosphorus predicted higher iPTH levels, even in the adjusted model. However, lower dietary phosphorus and fiber intake were predictors of higher iPTH levels, including in the adjusted model. Our results bring new data to the relationship between dietary intake and iPTH values. Despite higher serum phosphorus being observed in patients with HPTH, an opposite association was noted regarding dietary phosphate and fiber.

## 1. Introduction

Hyperparathyroidism (HPTH) is a common complication in patients undergoing maintenance hemodialysis (HD). Considering the Kidney Disease Improving Global Outcomes (KDIGO) targets, HPTH is present when intact parathyroid hormone (iPTH) serum levels are above 585 pg/mL (9 times normal) [1]. This pathology is caused by a disturbance in the mineral metabolism between calcium, phosphorus and vitamin D, and is consequently associated with clinical complications such as bone and mineral alterations, leading to osteodystrophy and cardiovascular abnormalities [2,3,4,5,6]. Higher levels of iPTH have been shown to increase calcification and thrombotic events, leading to a reduced health-related quality of life and increased mortality risk among patients with chronic kidney disease (CKD) [7,8]. From the nutritional status point of view, HPTH is associated with anorexia, increased protein catabolism and hyperlipidemia [9,10]. An early and appropriate management of mineral and bone markers is the most important intervention [11]. In addition to pharmacological treatment, it is important to frequently review serum phosphorus and calcium levels, as well as serum iPTH, to maintain them in line with the recommended KDIGO targets [1].

Although there is little evidence on the relationship between iPTH levels and nutritional status, some recent research suggests that high iPTH serum levels may lead to a poorer nutritional status in HD patients [9,12,13,14]. Protein-energy wasting (PEW) is a common complication (with a prevalence between 30 and 50%) that also affects morbidity and mortality in patients undergoing HD [15,16,17,18,19]. Its causes are multifactorial [20], as low energy and protein intake, dietary restrictions, dialysis-induced nutrient losses, increased inflammation and a loss of appetite can lead to this condition in HD patients [17,18,19,20,21]. Clinical and experimental data suggest that HPTH may increase resting energy expenditure (REE) and consequently lead to PEW and muscle wasting [9]. Ribeiro et al. found that a decreased appetite in patients with HPTH was associated with poorer nutritional status [10]. This finding may be explained by the fact that iPTH is a uremic hormone and leads to a loss of appetite and anorexia [13]. In addition, an epidemiological study supports HPTH as a cause of wasting and found a 12-month weight loss associated with HPTH levels [13].

However, there is no consensus on the relationship between weight loss and energy intake in HPTH patients, as there are studies that have found no statistical differences regarding energy intake when comparing between patients with HPTH and a control group [15,22,23]. Moreover, some data may link HPTH and nutritional impairment, since a few studies have shown that nutritional markers tend to be optimized in patients after parathyroidectomy [9,24,25].

Despite evidence on the relationship between iPTH levels and nutritional status being scarce, intervention frequently focuses on the appropriate management of mineral and bone markers, with the decrease in serum and dietary phosphorus being two of the targets. Therefore, this study aimed to investigate the association between iPTH, serum phosphorus levels and dietary intake.

## 2. Results

Of the whole sample (*n* = 561), 58.8% were men and 31.6% presented diabetes mellitus. The patients’ mean age was 68 ± 14 years, their median HD vintage was 65 (IQR: 43–106) months and their mean iPTH was 445 ± 388 pg/mL.

The patients were divided into three groups depending on their iPTH levels:

- iPTH < 130 pg/mL—13.9% (*n* = 81);
- iPTH between 130–585 pg/mL—59.3% (*n* = 345);
- iPTH >585 pg/mL—23.2% (*n* = 135).

The prevalence of patients with prescribed phosphate binders was 49.4% in the group with iPTH < 130 pg/mL; 43.5% in the group with iPTH between 130 and 585 pg/mL; and 54.1% in the group with iPTH > 585 pg/mL.

The differences observed between the three groups of patients regarding their clinical parameters, body composition and dietary intake are presented in Table 1.

No statistically significant differences were observed between the other vitamins analyzed (vitamin A, other B vitamins, vitamin C, D, E, K).

We observed a positive correlation between iPTH and serum calcium, serum phosphorus, calcium–phosphorus product and caffeine intake, and a negative correlation with age and intakes of calcium, phosphorus, fiber, folate and riboflavin. These results are presented in Table 2.

The linear regression models, adjusted for the variables of interest, showed that higher serum phosphorus levels predicted higher iPTH levels (Table 3). On the other hand, higher dietary intakes of phosphorus and fiber predicted lower iPTH levels (Table 4).

## 3. Discussion

In this multicenter, cross-sectional study, we observed a clear relationship between iPTH and serum phosphorus, dietary phosphorus and fiber intake in a multivariate model adjusted for potential confounders. Our data revealed that patients with HPTH presented a higher HD vintage of 73 (47–119) months and were younger (64 ± 15 years), facts that have already been observed by other authors [22,26].

Considering KDIGO targets [1], the prevalence of patients with HPTH in our sample was 23.2%. Other authors have reported higher prevalences when using different upper limits as their reference for HPTH. Lau et al. found that >80% of patients exhibited a serum iPTH > 150 pg/mL [27], whereas dialysis units reported 62% of patients had secondary HPTH (≥600 pg/mL) in 2014 [28].

It is well known that iPTH contributes to bone remodeling; therefore, altered levels can contribute to osteodystrophy. This association between HPTH and increased serum calcium and phosphorus levels is well documented, as altered iPTH levels are a consequence of renal function loss. When renal function decreases, phosphorus and uremic toxins accumulate in the blood. Consequently, phosphate retention stimulates the synthesis and release of phosphaturic hormones, including iPTH and FGF-23. Moreover, the increase in iPTH leads to a higher bone turnover which, in turn, increases phosphorus release from the bones, contributing to hyperphosphatemia [29]. However, there is still little evidence regarding HPTH and its related dietary factors.

Regarding the relationship between iPTH and diet, it is known that food intake interrupts its secretion, causing the pattern of iPTH release to change from continuous to intermittent, exerting a net bone anabolic activity [30]. On the other hand, starvation leads to hypocalcemia, which in turn leads to the continuous production of iPTH, which stimulates bone resorption, causing calcium release [31]. Despite the existing studies establishing the relationship between HPTH, a deterioration of the nutritional status [14] and anorexia [10,13], which leads to a reduced energy intake and weight loss, we did not observe any statistically significant differences regarding energy intake or body composition among the three groups of patients.

The habitual aim of the nutrition therapy in HPTH is to adjust food intake to maintain phosphorus levels within the target values. However, our data revealed that patients with HPTH were those with the lowest phosphorus intake, despite their higher serum phosphorus levels. Furthermore, our data showed that higher dietary phosphorus intake predicted lower iPTH levels, including in the model adjusted for potential confounders. On the one hand, phosphate metabolism and its reabsorption by the kidney is regulated via an interaction between the kidney, bone and gut, controlled by a cross-regulating endocrine network comprising iPTH, vitamin D, FGF-23 and Klotho protein. On the other hand, the bioavailability and absorption of dietary phosphorus varies depending on its food sources, which we did not differentiate between in the present study; it is well known that the phosphorus present in animal foods is much more easily absorbed compared to phosphorus from plant sources [32]. Furthermore, phosphate per se acts as a prebiotic that influences the growth of microbes [33,34,35], consequently, the reduction in dietary phosphate absorption and phosphate binders may also alter the gut microbiota [34]. In summary, nutritional intervention in patients with HPTH and hyperphosphatemia should not focus on restricting phosphorus intake but instead on choosing the most adequate food options, taking phosphorus bioavailability into account.

It has been described that CKD alters the gut microbiota, contributing to an increase in indoles and phenols, which are transformed, leading to the production of indoxyl sulfate and p-cresyl sulfate, respectively, which are associated, among other factors, with mineral and bone disorders in CKD patients. As these molecules are mainly protein-bound, the HD treatment itself does not eliminate them effectively [35]. Despite that, the fiber intake was below the recommended value (25–30 g/day) [36] in all groups, and those with HPTH presented the lowest intakes, of 17.5 g/day. In fact, higher dietary fiber intake predicted lower iPTH levels. There are several healthy food choices such as whole grains and pulses that are rich in fiber and also in phosphorus. As already mentioned, one of the aims of nutrition therapy in HPTH when high phosphorus levels are present is to adjust the dietary phosphorus intake, but high-fiber foods from plant sources should not be restricted despite their higher phosphorus content. The consumption of these foods contributes to an increased daily fiber intake, playing an important role in the microbiota balance. Furthermore, phosphorus bioavailability from vegetable sources is lower, so the impact of these foods on serum phosphorus will also be low [37]. Nonetheless, dietary fiber is the key substrate for the microbial growth of bacteria living in the gut [38]. Therefore, individualized approaches, through dietary modifications promoting the symbiotic status of the gut microbiota in CKD, mainly by increasing fiber intake and the impact on mineral and bone disorders, should be analyzed in future studies.

To the best of our knowledge, this is the first study analyzing the relationship between iPTH and complex B vitamins’ intake in HD patients. We observed that the lowest riboflavin and folate consumptions were registered in those patients with iPTH levels > 585 pg/mL, which could be related to the lower fiber intake also observed in this group of patients, as some of the food groups rich in fiber also present increased amounts of vitamins, such as fruits, vegetables and pulses. However, the dietary reference intake (DRI) for folate of 400 mcg/day [39] was not achieved in any of the groups. The same was not observed for riboflavin, as all the groups achieved the DRI of 1.3 mg/day for men and 1.1 mg/day for women [39].

Little evidence exists on the impact of high coffee intake (around 300 mg of caffeine) on low bone mineral density [40,41,42], especially when low intakes of calcium occur, as caffeine induces calcium release from the bone [43]. Our study revealed that patients with the highest intakes of caffeine (79 mg/day) were those in the group of patients with higher levels of iPTH (>585 pg/mL). Lu et al. speculated that caffeine might affect bone quality through the modulation of iPTH secretion, by eventually suppressing it [44]. However, this iPTH suppression effect was observed only with high caffeine serum concentrations (50 μM). Considering that the results obtained in the three groups showed a low mean caffeine intake, we believe that we did not observe the same association, as the doses of caffeine consumed observed in our study were far from the values that may be implicated in CKD-MBD.

The elevated number of participants enrolled, and the fact that 37 dialysis units around the country were included, as well as our new insights on dietary intake and its relationship with iPTH levels, are the clear strengths of this study. In terms of limitations, we point out the cross-sectional design and the fact that the data about dietary intake were obtained through retrospective questionnaires, where answers depend on the precision of patients’ recollections, as well as the lack of differentiation between different sources of dietary phosphorus.

## 4. Materials and Methods

### 4.1. Study Design and Setting

This cross-sectional, multicenter, observational study was conducted on 561 patients receiving HD treatment at thirty-seven dialysis centers located in Portugal.

### 4.2. Sample Size

Of the 4600 patients receiving in-center HD, 561 patients were eligible; patients meeting the inclusion criteria were randomly selected equally from each dialysis center.

### 4.3. Inclusion and Exclusion Criteria

Inclusion criteria for participation in this study were: age ≥ 18 years, four hour in-center dialysis treatment 3 times a week for ≥15 months (with an online hemodiafiltration technique), agreement to participate and the signing an informed consent. In most of the studies including HD subjects, a dialysis vintage of 3 months is defined. As the Food Frequency Questionnaire (FFQ) reports eating habits from the 12 months before its application, we considered a minimum dialysis vintage of 15 months.

All patients were dialyzed with high-flux membranes (Helixone^®^, Fresenius^®^ Medical Care, Bad Homburg, Germany) and ultrapure water in accordance with the criteria of ISO regulation 13,959:2009—water for hemodialysis and related therapies. Furthermore, participants had an initial nutrition consultation when they started HD treatment, where they received nutritional recommendations according to the current guidelines. Patients were not selected if they presented a poor understanding of the Portuguese language, severe neurological or mental disorders, active neoplasic disease, major amputations (lower/upper extremities), enteral or parenteral feeding, severe alcohol or drug addiction, hepatitis C with viral replication, liver disease, or if they were taking immunosuppressive or corticoid medication.

### 4.4. Data Analysis

Demographic, biochemical (including iPTH), anthropometric, dialysis treatment data and the etiology of the CKD were obtained from the dialysis unit database in the same month as the face-to-face interviews, at the beginning of the study. The blood collected for biochemical analysis was taken before the mid-week HD treatment. All blood tests were analyzed using identical methods in different external laboratories. Prescribed phosphate binders were also registered and those included calcium carbonate, sevelamer carbonate, sucroferric oxyhydroxide and calcium acetate/magnesium carbonate.

### 4.5. Body Composition

Body composition assessment was carried out on the 561 eligible patients and included the lean tissue index (Kg/m^2^) (LTI), fat tissue index (Kg/m^2^) (FTI), body cell mass (Kg) (BCM), % relative overhydration/extracellular water measured pre-dialysis, total body water (L) (TBW), extracellular water (L) (ECW) and intracellular water (L) (ICW). These parameters were analyzed via bioimpedance spectroscopy with a Body Composition Monitor^®^ (BCM) (Fresenius Medical Care Deutschland GmbH, Bad Homburg, Germany). The BCM takes measurements at 50 frequencies in a range of 5–1000 KHz. Measurements were collected approximately 30 min before the midweek HD session, using 4 conventional electrodes for each patient (two on the hand and two on the foot, contralateral to the vascular access). For the assessment, patients were lying in the supine position. Two advanced physiological models are used in the BCM to obtain clinically relevant parameters:

- A volume model that describes the electrical conductance in a cell suspension, allowing for the calculation of the TBW, ECW and ICW [45];
- A body composition model that divides body mass into 3 compartments: overhydration, lean tissue, and adipose tissue [46].

All parameters were validated against the gold standard reference methods used in several studies with more than 500 patients and healthy controls. The measurements presented a quality index greater than 95%.

### 4.6. Food Frequency Questionnaire (FFQ)

Dietary assessment was performed by a trained dietitian with a semi-quantitative FFQ in a face-to-face interview during HD treatment. This tool was developed and has already been validated for the Portuguese population [47,48]. It presents 95 food items, 9 categories of frequencies (from “never or less than once a month” to “six or more times a day”), and a section with predetermined average portions. A visual aid was provided to patients through an illustrated book with 131 pictures of the servings. The frequency of intake and average servings of each food were registered considering the patients’ diet over the past 12 months.

To estimate food consumption, the reported frequency of each food was multiplied by its respective portion (in grams) and, in some foods consumed at specific times of the year, by a seasonal variation factor. This questionnaire provides information on the average daily amount of macro and micronutrients that patients consume. The conversion of the different food items into nutrients was performed using the Food Processor Plus software (ESHA Research, Salem, Oregon, version 11.6), which contained nutritional data from the United States Department of Agriculture and then adapted it to traditional Portuguese food. The data on Portuguese food nutrients were added to the original database using the Portuguese food composition table [49]. During data analysis, foods with an average consumption ≤5 g/day were excluded.

### 4.7. Statistical Analysis

For the analysis, continuous variables were presented as mean  ± standard deviation (SD) or as median and interquartile ranges (IQR), and categorical variables were presented as a frequency (percentages). The distribution of the data was tested with a Kolmogorov–Smirnov test.

For the analysis, eligible patients were divided into 3 groups, depending on their iPTH levels: (a) iPTH < 130, (b) iPTH between 130 and 585 and (c) iPTH > 585 pg/mL [1].

A Spearman correlation was used to analyze the correlation between iPTH and the variables of interest. Mean differences were assessed with a one-way ANOVA for normally distributed variables and with the Kruskal–Wallis test for variables that were not normally distributed. The Chi-square test was used to analyze phosphate binder prescriptions across the 3 groups of patients.

The multiple linear regression model that included iPTH as the dependent variable and serum phosphorus was adjusted for age, gender, energy intake, dietary phosphorus intake, dialysis adequacy (Kt/V) and dialysis vintage, whereas the multiple linear regression model that included iPTH as the dependent variable and dietary phosphorus intake was adjusted for age, gender, serum phosphorus, dialysis adequacy (Kt/V), dialysis vintage and phosphate binders.

Statistical analysis was run using the IBM SPSS software (version 28.0; IBM SPSS, Inc., Chicago, IL, USA) and a *p*-value < 0.05 was considered statistically significant.

## 5. Conclusions

Our results bring new data to the relationship between dietary intake and iPTH values. A clear association relationship between iPTH, serum phosphorus, dietary phosphorus and fiber consumption was observed in this study. Despite higher serum phosphorus being observed in patients with HPTH, an opposite association was noted regarding dietary phosphate and fiber, as lower intakes of these predicted higher iPTH values. Moreover, a poorer dietary intake, considering riboflavin and folate, was observed in the HPTH group.

In summary, nutritional intervention in patients with HPTH and hyperphosphatemia should not focus on restricting dietary phosphorus from all food sources but rather on choosing the most adequate food options considering the bioavailability of phosphorus, therefore avoiding the phosphate additives that are often present in processed foods and fast food.

It is crucial to have an individualized nutritional approach that prioritizes fiber intake to promote the symbiotic status of the gut microbiota in these patients, as this could contribute to attenuate their bone mineral disease.

## Figures and Tables

**Table 1:** Differences between iPTH groups.

Parameter	iPTH < 130 pg/mL	iPTH 130–585 pg/mL	iPTH > 585 pg/mL	*p*
Age (years)	68 ± 14	69 ± 13 β	64 ± 15 β	**0.002**
HD vintage (months)	62 (41–105)	62 (42–101) β	73 (47–119) β	**0.021**
Kt/V	1.8 (1.5–1.9)	1.7 (1.5–1.9)	1.7 (1.4–2.0)	0.985
Dry weight (kg)	67.0 (59.5–77.9)	69.5 (61.5–78.2)	70.0 (61–81.5)	0.325
Serum phosphorus (mg/dL)	4.2 ± 1.4	4.2 ± 1.1 β	4.6 ± 1.0 β	**0.005**
Serum calcium (mg/dL)	8.9 ± 1.0	8.9 ± 0.6 β	9.1 ± 0.8 β	**0.027**
Calcium–phosphorus product	37.8 ± 15.2 β	37.6 ± 10.7 ¥	42.0 ± 10.5 β¥	**<0.001**
Potassium (mEq/L)	5.3 ± 0.7	5.3 ± 0.7	5.3 ± 0.6	0.760
Albumin (g/dL)	4.0 (3.8–4.2) β	4.0 (3.8–4.2) ¥	4.1 (4.0–4.3) β¥	**0.016**
C-reactive protein (mg/L)	10.9 ± 14.5	9.8 ± 12.6	12.8 ± 18.7	0.662
Body mass index (kg/m^2^)	24.9 (22.1–28.3)	25.8 (22.8–29.4)	26.0 (22.6–29.1)	0.747
Lean tissue index (kg/m^2^)	12.3 (10.5–14.4)	12.1 (10.7–13.6)	12.9 (10.9–14.7)	0.090
Fat tissue index (kg/m^2^)	12.0 (9.4–15.6)	13.7 (9.7–17.6)	12.9 (9.0–16.5)	0.250
Energy intake (kcal)	1896 (1395–2341)	1829 (1482–2361)	1722 (1390–2173)	0.125
Protein intake (g/day)	75.4 (60.8–102.7)	76.7 (61.1–99.9)	72.9 (52.8–93.0)	0.103
Carbohydrate intake (g/day)	235 (172–317)	247 (191–300)	224 (172–278)	0.073
Fat intake (g/day)	57.9 (43.5–79.8)	57.7 (44.5–77.6)	54.9 (43.8–75.1)	0.516
Dietary fiber intake (g/day)	20.1 (13.9–27.6)	20.0 (14.4–26.6) β	17.5 (14.7–22.1) β	**0.047**
Calcium intake (mg/day)	687 (498–911)	680 (460–938)	615 (447–860)	0.119
Phosphorus intake (mg/day)	1089 (821–1387)	1093 (833–1414) β	986 (756–1286) β	**0.044**
Potassium intake (mg/day)	2453 (1786–3156)	2378 (1814–2967)	2215 (1758–2651)	0.077
Riboflavin (mg/day)	1.7 (1.3–2.0)	1.7 (1.3–2.1) β	1.5 (1.1–2.0) β	**0.031**
Folate (mcg/day)	220 (157–304) ¥	210 (160–279) β	192 (149–236) β¥	**0.011**
Caffeine (mg/day)	56 (15–81) ¥	45 (20–81) β	79 (32–86) β¥	**0.009**

HD, hemodialysis; Kt/V, dialysis adequacy; β and ¥, groups with statistically significant differences established through Bonferroni post hoc tests. Bold numbers highlight statistical significance.

**Table 2:** Spearman correlations between iPTH and parameters of interest.

	r	*p*
Age (years)	−0.145	**<0.001**
Serum calcium (mg/dL)	0.114	**0.007**
Serum phosphorus (mg/dL)	0.141	**<0.001**
Calcium–phosphorus product	0.157	**<0.001**
Albumin (g/dL)	0.090	0.033
C-reactive protein (mg/L)	0.088	0.339
Energy intake (Kcal/day)	−0.082	0.052
Protein intake (g/day)	−0.083	0.050
Carbohydrate intake (g/day)	−0.082	0.052
Fat intake (g/day)	−0.050	0.233
Fiber intake (g/day)	−0.086	**0.042**
Calcium intake (mg/day)	−0.099	**0.008**
Phosphorus intake (mg/day)	−0.112	**0.019**
Vitamin D intake (mcg/day)	−0.058	0.168
Folate (mcg/day)	−0.120	**0.004**
Riboflavin (mg/day)	−0.109	**0.010**
Caffeine (mg/day)	0.097	**0.022**

Bold numbers highlight statistical significance.

**Table 3:** Linear regression between iPTH and serum phosphorus.

Dependent Variable	Independent Variable	*B*	95% CI_a_ *	*p*
**Intact PTH**	Serum Phosphorus	39.6	5.9–73.4	0.022

* Multivariate model adjusted for age, gender, phosphorus intake, dialysis adequacy (Kt/V), dialysis vintage and phosphate binders. Bold numbers/letters highlight statistical significance.

**Table 4:** Linear regression between iPTH and phosphorus intake—dependent variable iPTH.

Dependent Variable	Independent Variable	*B*	95% CI_a_ *	*p*
**Intact PTH**	Phosphorus intake	−0.090	−0.17–(−0.007)	0.033
**Intact PTH**	Fiber intake	−3.713	−7.35–(−0.046)	0.047

* Multivariate model adjusted for age, gender, serum phosphorus, dialysis adequacy (Kt/V), dialysis vintage and phosphate binders. Bold numbers/letters highlight statistical significance.

## Data Availability

The data presented in this study are available on request from the corresponding author. The data are not publicly available due to privacy issues.

## References

[1] Kidney Disease: Improving Global Outcomes (KDIGO) CKD-MBD Update Work Group (2017). Kidney Disease: Improving Global Outcomes CKDMBDUWG. KDIGO 2017 Clinical Practice Guideline Update for the Diagnosis, Evaluation, Prevention, and Treatment of Chronic Kidney Disease-Mineral and Bone Disorder (CKD-MBD). Kidney Int. Suppl..

[2] Dukkipati R., Kovesdy C.P., Colman S., Budoff M.J., Nissenson A.R., Sprague S.M., Kopple J.D., Kalantar-Zadeh K. (2010). Association of relatively low serum parathyroid hormone with malnutrition-inflammation complex and survival in maintenance hemodialysis patients. J. Ren. Nutr..

[3] Mizobuchi M., Ogata H., Koiwa F. (2019). Secondary Hyperparathyroidism: Pathogenesis and Latest Treatment. Ther. Apher. Dial..

[4] Brandenburg V., Ketteler M. (2022). Vitamin D and Secondary Hyperparathyroidism in Chronic Kidney Disease: A Critical Appraisal of the Past, Present, and the Future. Nutrients.

[5] Feroze U., Molnar M.Z., Dukkipati R., Kovesdy C.P., Kalantar-Zadeh K. (2011). Insights into nutritional and inflammatory aspects of low parathyroid hormone in dialysis patients. J. Ren. Nutr..

[6] Fujii H. (2018). Association between Parathyroid Hormone and Cardiovascular Disease. Ther. Apher. Dial..

[7] Tentori F., Wang M., Bieber B.A., Karaboyas A., Li Y., Jacobson S.H., Andreucci V.E., Fukagawa M., Frimat L., Mendelssohn D.C. (2015). Recent changes in therapeutic approaches and association with outcomes among patients with secondary hyperparathyroidism on chronic hemodialysis: The DOPPS study. Clin. J. Am. Soc. Nephrol..

[8] Floege J., Kim J., Ireland E., Chazot C., Drueke T., de Francisco A., Kronenberg F., Marcelli D., Passlick-Deetjen J., Schernthaner G. (2011). Serum iPTH, calcium and phosphate, and the risk of mortality in a European haemodialysis population. Nephrol. Dial. Transplant..

[9] Cuppari L., de Carvalho A.B., Avesani C.M., Kamimura M.A., Dos Santos Lobao R.R., Draibe S.A. (2004). Increased resting energy expenditure in hemodialysis patients with severe hyperparathyroidism. J. Am. Soc. Nephrol..

[10] Ribeiro M., Vogt B.P., Vannini F.C.D., Caramori J.C.T. (2019). Role of parathyroid hormone in anorexia on maintenance hemodialysis patients. Clin. Nutr. ESPEN.

[11] Friedl C., Zitt E. (2018). Role of etelcalcetide in the management of secondary hyperparathyroidism in hemodialysis patients: A review on current data and place in therapy. Drug Des. Devel. Ther..

[12] Kir S., Komaba H., Garcia A.P., Economopoulos K.P., Liu W., Lanske B., Hodin R.A., Spiegelman B.M. (2016). PTH/PTHrP Receptor Mediates Cachexia in Models of Kidney Failure and Cancer. Cell Metab..

[13] Komaba H., Zhao J., Yamamoto S., Nomura T., Fuller D.S., McCullough K.P., Evenepoel P., Christensson A., Zhao X., Alrukhaimi M. (2021). Secondary hyperparathyroidism, weight loss, and longer term mortality in haemodialysis patients: Results from the DOPPS. J. Cachexia Sarcopenia Muscle.

[14] Disthabanchong S., Vantanasiri K., Khunapornphairote S., Chansomboon P., Buachum N., Saeseow S. (2022). Severe hyperparathyroidism is associated with nutritional impairment in maintenance hemodialysis patients. Front. Nutr..

[15] Komaba H., Fukagawa M. (2018). Secondary Hyperparathyroidism and Protein-Energy Wasting in End-Stage Renal Disease. Ther. Apher. Dial..

[16] Himmelfarb J., Vanholder R., Mehrotra R., Tonelli M. (2020). The current and future landscape of dialysis. Nat. Rev. Nephrol..

[17] Majlessi A., Burton J.O., March D.S. (2022). The effect of extended hemodialysis on nutritional parameters: A systematic review. J. Nephrol..

[18] Sahathevan S., Khor B.H., Ng H.M., Gafor A.H.A., Daud Z.A.M., Mafra D., Karupaiah T. (2020). Understanding Development of Malnutrition in Hemodialysis Patients: A Narrative Review. Nutrients.

[19] Cano N. (2001). Hemodialysis, inflammation and malnutrition. Nefrologia.

[20] Carrero J.J., Stenvinkel P., Cuppari L., Ikizler T.A., Kalantar-Zadeh K., Kaysen G., Mitch W.E., Price S.R., Wanner C., Wang A.Y.M. (2013). Etiology of the protein-energy wasting syndrome in chronic kidney disease: A consensus statement from the International Society of Renal Nutrition and Metabolism (ISRNM). J. Ren. Nutr..

[21] raterol Torres F., Molina M., Soler-Majoral J., Romero-González G., Rodríguez Chitiva N., Troya-Saborido M., Socias Rullan G., Burgos E., Paúl Martínez J., Urrutia Jou M. (2022). Evolving Concepts on Inflammatory Biomarkers and Malnutrition in Chronic Kidney Disease. Nutrients.

[22] Rezende L.T., Cuppari L., Carvalho A.B., Canziani M.E., Manfredi S.R., Cendoroglo M., Sigulem D.M., Draibe S.A. (2000). Nutritional status of hemodialysis patients with secondary hyperparathyroidism. Braz. J. Med. Biol. Res..

[23] Campos S.R., Gusmao M.H., Almeida A.F., Pereira L.J., Sampaio L.R., Medeiros J.M. (2012). Nutritional status and food intake of continuous peritoneal dialysis patients with and without secondary hyperparathyroidism. J. Bras. Nefrol..

[24] Khajehdehi P., Ali M., Al-Gebory F., Henry G., Bastani B. (1999). The effects of parathyroidectomy on nutritional and biochemical status of hemodialysis patients with severe secondary hyperparathyroidism. J. Ren. Nutr..

[25] Yasunaga C., Nakamoto M., Matsuo K., Nishihara G., Yoshida T., Goya T. (1999). Effects of a parathyroidectomy on the immune system and nutritional condition in chronic dialysis patients with secondary hyperparathyroidism. Am. J. Surg..

[26] Chertow G.M., Plone M., Dillon M.A., Burke S.K., Slatopolsky E. (2000). Hyperparathyroidism and dialysis vintage. Clin. Nephrol..

[27] Lau W.L., Obi Y., Kalantar-Zadeh K. (2018). Parathyroidectomy in the Management of Secondary Hyperparathyroidism. Clin. J. Am. Soc. Nephrol..

[28] National Kidney Foundation (2003). K/DOQI clinical practice guidelines for bone metabolism and disease in chronic kidney disease. Am. J. Kidney Dis..

[29] Filipska I., Winiarska A., Knysak M., Stompor T. (2021). Contribution of Gut Microbiota-Derived Uremic Toxins to the Cardiovascular System Mineralization. Toxins.

[30] Pacifici R. (2021). Role of Gut Microbiota in the Skeletal Response to PTH. J. Clin. Endocrinol. Metab..

[31] Kermgard E., Chawla N.K., Wesseling-Perry K. (2021). Gut microbiome, parathyroid hormone, and bone. Curr. Opin. Nephrol. Hypertens..

[32] D’Alessandro C., Piccoli G.B., Cupisti A. (2015). The “phosphorus pyramid”: A visual tool for dietary phosphate management in dialysis and CKD patients. BMC Nephrol..

[33] Ye G., Yang W., Bi Z., Huang L., Liu F. (2021). Effects of a high-phosphorus diet on the gut microbiota in CKD rats. Ren. Fail..

[34] Favero C., Carriazo S., Cuarental L., Fernandez-Prado R., Goma-Garces E., Perez-Gomez M.V., Ortiz A., Fernandez-Fernandez B., Sanchez-Niño M.D. (2021). Phosphate, Microbiota and CKD. Nutrients.

[35] Biruete A., Cross T.L., Allen J.M., Kistler B.M., de Loor H., Evenepoel P., Fahey G.C., Bauer L., Swanson K.S., Wilund K.R. (2021). Effect of Dietary Inulin Supplementation on the Gut Microbiota Composition and Derived Metabolites of Individuals Undergoing Hemodialysis: A Pilot Study. J. Ren. Nutr..

[36] Ikizler T.A., Burrowes J.D., Byham-Gray L.D., Campbell K.L., Carrero J.J., Chan W., Fouque D., Friedman A.N., Ghaddar S., Goldstein-Fuchs D.J. (2020). KDOQI Clinical Practice Guideline for Nutrition in CKD: 2020 Update. Am. J. Kidney Dis..

[37] Byrne F., Gillman B., Palmer B., Kiely M., Eustace J., Kearney P., Davidson F., Shiely F. (2021). The effect of dietary phosphorus load and food matrix on postprandial serum phosphate in hemodialysis patients: A pilot study. HRB Open Res..

[38] Camerotto C., Cupisti A., D’Alessandro C., Muzio F., Gallieni M. (2019). Dietary Fiber and Gut Microbiota in Renal Diets. Nutrients.

[39] Board IoMFaN (1998). Dietary Reference Intakes: Thiamin, Riboflavin, Niacin, Vitamin B6, Folate, Vitamin B12, Pantothenic Acid, Biotin, and Choline.

[40] Dew T.P., Day A.J., Morgan M.R. (2007). Bone mineral density, polyphenols and caffeine: A reassessment. Nutr. Res. Rev..

[41] Kiel D.P., Felson D.T., Hannan M.T., Anderson J.J., Wilson P.W. (1990). Caffeine and the risk of hip fracture: The Framingham Study. Am. J. Epidemiol..

[42] Hallstrom H., Wolk A., Glynn A., Michaelsson K. (2006). Coffee, tea and caffeine consumption in relation to osteoporotic fracture risk in a cohort of Swedish women. Osteoporos. Int..

[43] Lerner U.H., Mellstrom D. (1992). Caffeine has the capacity to stimulate calcium release in organ culture of neonatal mouse calvaria. Calcif. Tissue Int..

[44] Lu M., Farnebo L.O., Branstrom R., Larsson C. (2013). Inhibition of parathyroid hormone secretion by caffeine in human parathyroid cells. J. Clin. Endocrinol. Metab..

[45] Moissl U.M., Wabel P., Chamney P.W., Bosaeus I., Levin N.W., Bosy-Westphal A., Korth O., Müller M.J., Ellegård L., Malmros V. (2006). Body fluid volume determination via body composition spectroscopy in health and disease. Physiol. Meas..

[46] Chamney P.W., Wabel P., Moissl U.M., Muller M.J., Bosy-Westphal A., Korth O., Fuller N.J. (2007). A whole-body model to distinguish excess fluid from the hydration of major body tissues. Am. J. Clin. Nutr..

[47] de Moura Lopes C.M. (2000). Reprodutibilidade e Validação de Um Questionário Semi-Quantitativo de Frequência Alimentar. Alimentação e Enfarte Agudo do Miocárdio—Estudo Caso-Controlo de Base Comunitária.

[48] Lopes C., Aro A., Azevedo A., Ramos E., Barros H. (2007). Intake and adipose tissue composition of fatty acids and risk of myocardial infarction in a male Portuguese community sample. J. Am. Diet Assoc..

[49] Ferreira F.G.M. (1985). Tabela de Composição de Alimentos Portugueses.

